# Determination of Ursolic Acid in Extracts From *Ligustri lucidum* Fruit Using an Electrochemical Method

**DOI:** 10.3389/fchem.2020.00444

**Published:** 2020-05-27

**Authors:** Yingpu Feng, Zheng Wei, Junping Zhang

**Affiliations:** ^1^Centre of Cerebrovascular, Zhengzhou University People's Hospital, Zhengzhou, China; ^2^Centre of Cerebrovascular, Henan Provincial People's Hospital, Zhengzhou, China; ^3^Department of Traditional Chinese Medicine, Henan Academy Institute of Traditional Chinese Medicine, Zhengzhou, China

**Keywords:** electrochemical sensor, Pt nanoparticles, voltammetry, nanocomposite, boron nitride nanosheets, *Ligustri lucidum*, ursolic acid, biosensor

## Abstract

In this work, we reported a facile wet chemical method for depositing Pt nanoparticles on the surface of boron nitride nanosheets (BNNS-Pt NPs). The deposited nanocomposite was applied for glassy carbon electrode surface modification. The modified electrode was then used for detecting ursolic acid (UA). The results indicate that the BNNS-Pt NPs exhibited excellent electrocatalytic activity toward UA oxidation compared with that of the bare glassy carbon electrode (GCE) and Pt NPs/GCE. The UA oxidation currents is linearly related its concentration from 1 to 1,200 pM. The limit of detection can be calculated to be 0.5 pM. In addition, the UA sensor was also successfully used for the determination of UA in *Ligustri lucidum* fruit samples.

## Introduction

Privet fruit is the ripe fruit of the *Ligustrum lucidum* Ait plant (Hu et al., 2014). This fruit has the function of nourishing the liver and kidneys and improving eyesight according to the study of traditional Chinese medicine (Gao et al., 2015a). The benefits of privet fruit are gradual, but their long-term use can be effective. Modern clinical applications also include the treatment of optic neuritis, leukocyte reduction, chronic hepatitis, hyperlipidemia, coronary heart disease, hypertension, children's poisoning, hearing loss, neurasthenia, facial paralysis, and hair loss (Gao et al., 2007; Pang et al., 2015). A study found that *L. lucidum* fruit contains a high quantity of ursolic acid (UA). UA is a pentacyclic triterpenoid identified in many herbs. UA has many biological effects, such as acting as a sedative, having anti-inflammatory, antibacterial, anti-diabetes, and anti-ulcer effects, and reducing blood sugar (Li et al., 2019). UA is also widely used as a raw material for medicine and cosmetics because of its antioxidant function. The clinical uses of UA demonstrate significantly decreased serum aminotransferase and alanine aminotransferase, alleviated jaundice, increased appetite, suppressed fibrosis and recovered liver function, with the advantages of fast and stable effects and short treatment course (Hussain et al., 2017).

Ursolic acid (5~20 mg/kg) can treat rat liver toxicity (Saraswat et al., 2000; Saraswati et al., 2013). Pretreatment with UA can significantly improve the survival rate of rat hepatocytes and can provide anti-cholestasis effects (Chai et al., 2015). Both the flow and the contents of bile increased accordingly. According to the mechanism of CC1, the mechanism of UA in the treatment of damaged hepatocytes may be similar to that of the isomers of oleanolic acid, which is to protect and stabilize the biofilm system of the liver cell membrane and organelles (Yan et al., 2010). The change was evident, and active transport functions returned to normal. The distribution of mobile ions and water inside and outside the cells recover consequently, which restores the regeneration function and promotes the repair of hepatic centrilobular necrosis of the liver cells (Alam et al., 2018). The study found that UA plays an important role in the treatment of viral hepatitis whether it is used alone or with other drugs in preparation for the treatment of viral hepatitis. Compared with oleanolic acid (Liu, 1995), 102 cases of acute hepatitis A and B were treated with UA with a dose of 102 and 60 mg/d; the average time of treatment was 21 days, and the cure rate was 89.3%. The curative effect of 100 cases treated with oleanolic acid (68%, *P* < 0.01) was better than that of the control group with the same treatment time. Clinical trials prove that UA shows a significant and rapid reduction in alanine transaminase, elimination of jaundice and recovery of liver function. After 3 weeks of treatment, the negative rate of 21 cases of HBeAg-positive patients was 61.9%. The negative rate of 21 cases of HBsAg positive patients was 42.8%, indicating that it also has a certain therapeutic effect on hepatitis B. In addition, Ramos-Hryb also confirmed that UA not only has the effect of treating viral hepatitis but also acts as an antidepressant; it contains UA perilla extract as a treatment for depression (Ramos-Hryb et al., 2017). UA causes almost no adverse reactions (Gharibi et al., 2018). Compared with western medicine, the antidepressant spectrum is narrow, while the toxicity and side effects are very minor (Machado et al., 2012). UA has the obvious advantages of decreasing recurrence and suicidal tendencies after drug use. The capsule made from the extract of *Ligustrum lucidum* leaves mainly composed of UA is primarily used for primary hyperlipidemia (Yuliang et al., 2015). Therefore, the development of a reliable method for UA determination is very important in clinical and pharmacological fields (Seo et al., 2018; Burhan et al., 2020; Demirkan et al., 2020).

Thus far, several methods have been developed for the determination of UA, including micellar electrokinetic capillary chromatography (Zhang et al., 2005), HPLC (Li et al., 2019), LC-MS (Novotny et al., 2003), capillary zone electrophoresis (Gao et al., 2015b) and UV spectroscopy (Pironi et al., 2018). These methods all require complicated sample preparation procedures and a sophisticated operation. An electrochemical method may be considered as an alternative for UA detection because of the high sensitivity, fast response and low detection limit (Karimi-Maleh et al., 2019a,b; Shamsadin-Azad et al., 2019; Tahernejad-Javazmi et al., 2019; Karimi-Maleh and Arotiba, 2020). However, electrochemical sensors have often been restricted by the high redox overpotential of target molecules. Therefore, electrode surface modification has been widely used for improving the sensing performance by triggering an electrocatalytic reaction (Baghayeri et al., 2014, 2018a,b; Orooji et al., 2019). A studyreported that BNNS can be used as a loading platform catalyst deposition and consequently applied as an excellent modifier for electrochemical sensing (Nam et al., 2018). In addition, BNNS showed superior dispersibility than graphene in aqueous conditions, which allows further construction (Azamat et al., 2015; Rajski et al., 2020).

In this study, we report a facial strategy for the *in situ* growth of Pt nanoparticles on BNNS without using additional reductants. The resulting BNNS-Pt nanocomposite has been modified on a glassy carbon electrode (GCE) for the electrocatalytic determination of UA. Meanwhile, the proposed approach has been successfully used for determining UA content in the *Ligustri lucidum* fruit extract.

## Experiments

The exfoliation of BN was performed according to the literature and contains two steps (Fu et al., 2017). In the first stage, NaOH (2.8434 g) and KOH (2.1566 g) were ground finely, and then BN powder (1 g) was added. The mixture was further ground into a homogeneous form and transferred to a 100 mL Teflon-lined stainless-steel autoclave. The autoclave was heated to 180°C, and the temperature was maintained for 2 h in an oven and then naturally cooled to room temperature. In the secondary stage, the solid product collected from the autoclave was dispersed in 300 mL of water. The dispersion was sonicated for 1 h using a sonic tip (90% of 600 W using 5 s on 2 s off pulsation). After a filtration process to remove hydroxide, the BN slurry was dispersed into 300 mL using 10 min of bath sonication before centrifugation. The BN dispersion was centrifuged at 2,000 rpm for 30 min to remove the aggregated material and thick flakes. The supernatant was collected as BNNS.

The BNNS-Pt NP composite was prepared by growing Pt NPs *in situ* on BNNS. Briefly, 0.2 mL of 2% H_2_PtCl_6_ was added into 25 mL BNNS (0.2 mg/mL). After the pH was adjusted to 10 using 0.1 M NaOH, the solution was stirred for 24 h at room temperature. After centrifugation and washing twice with water, the resulting BNNS-Pt NP composite was obtained and re-dispersed in 10 mL water for further use.

Scanning electron microscopy (SEM, QUANTA FEG250) was used for morphology characterization. XRD characterization was carried out with a BRUKER D8–Advance XRD system with Cu Kα radiation. All the electrochemical sensing and characterizations were conducted using a CHI 832 electrochemical workstation. All the experiments were performed at room temperature.

A glassy carbon electrode (GCE) was polished by 0.05 μm alumina dispersion and cleaned by water and ethanol. Then, 4 μL of catalyst dispersion (0.2 mg/mL) was dropped onto the GCE surface and dried naturally. The anti-interference study was carried out by square wave voltammetry (SWV) in the presence of UA and interference species. Triplicate measurements were recorded and the average values of current changes were calculated. Peanut shells and *Ligustri lucidum* (fruit) were purchased from a local traditional Chinese medical center. For the real sample preparation, the dried *Ligustri lucidum* (fruit) was grounded and then dispersed into 50 mL methanol with vigorous stirring for 12 h. Then, the dispersion was filtered through a 0.45 μm filter paper and diluted to 50 mL by PBS.

## Results and Discussion

**Figure 1:**
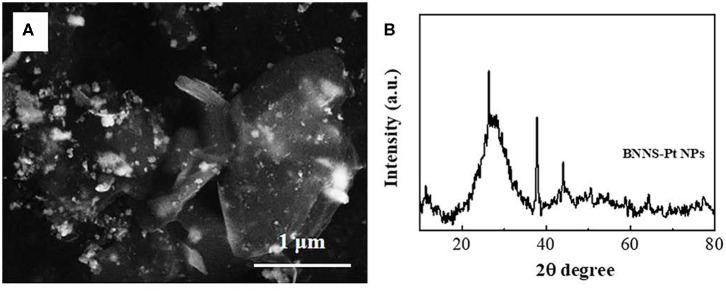
**(A)** SEM image and **(B)** XRD spectrum of BNNS-Pt NPs.

Figure 1A shows the SEM image of the synthesized BNNS-Pt NPs. It can be seen that the Pt NPs can be clearly differentiated from the BNNS surface by the contrast. The XRD patterns of the BNNS and BNNS-Pt NP composites are shown in Figure 1B. The characteristic peaks at 26.8°, 41.7°, 44.2°, 50.3°, 55.2°, 71.4°, and 76.1° can be assigned to the (002), (100), (101), (102), (004), (104), and (110) planes of the hexagonal phase of BN (JCPDS 34-0421) (Lim et al., 2013), respectively. The additional peaks at 38.5°, 44.7°, 64.4, and 77.6 in the XRD pattern of the BNNS-Pt NP composite are attributed to the (111), (200), (220), and (331) crystal face of face-centered cubic (fcc) Pt (JCPDS 65-2870) (Wang et al., 2014). The average size of the Pt NPs can be calculated to be 21 nm.

**Figure 2:**
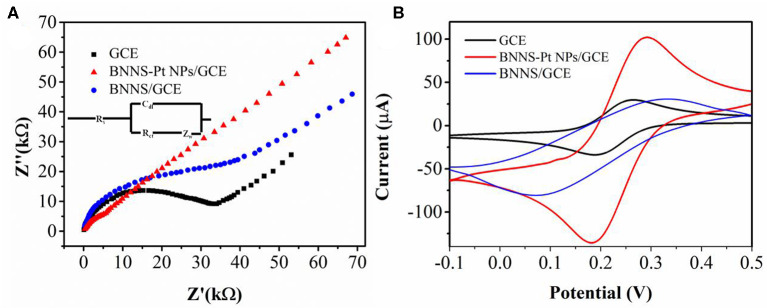
**(A)** Electrochemical impedance spectra and **(B)** CV profiles of bare GCE, BNNS/GCE, and BNNS-Pt NP/GCE in 0.1 M KCl containing 5 mM [Fe(CN)_6_]^3−/4−^.

The electrochemical properties of bare GCE and modified GCE were investigated using 5 mM [Fe(CN)_6_]^3−/4−^ in 0.1 M KCl by electrochemical impedance spectroscopy (EIS). As shown in Figure 2A, the EIS spectrum of BNNS/GCE shows a much larger semicircle than that of bare GCE due to the low electron transfer rate, indicating that BNNS hindered interfacial electron transfer. In contrast, BNNS-Pt NP/GCE shows much smaller semicircles, suggesting that the surface modification promotes the electron transfer rate on the GCE surface.

Cyclic voltammetry was used to further study the electrochemical properties of various electrodes (Figure 2B). A clear change was noticed in the voltammograms before and after catalyst modification. The electrochemical profiles of [Fe(CN)_6_]^3−/4−^ redox is largely suppressed with a larger peak to peak splitting, indicating that the insulation of the BNNS lowers the electron transfer rate, which agrees with the EIS result (Fu et al., 2018b). On the other hand, a clear improvement of the current intensity is noticed after the loading of Pt NPs. In addition, BNNS-Pt NPs/GCE displays an even higher response than Pt NPs/GCE, indicating that the loading of Pt NPs on BNNS surface can provide more electrocatalytic sites by exposing individual Pt NPs to the [Fe(CN)_6_]^3−/4−^ without agglomeration.

**Figure 3:**
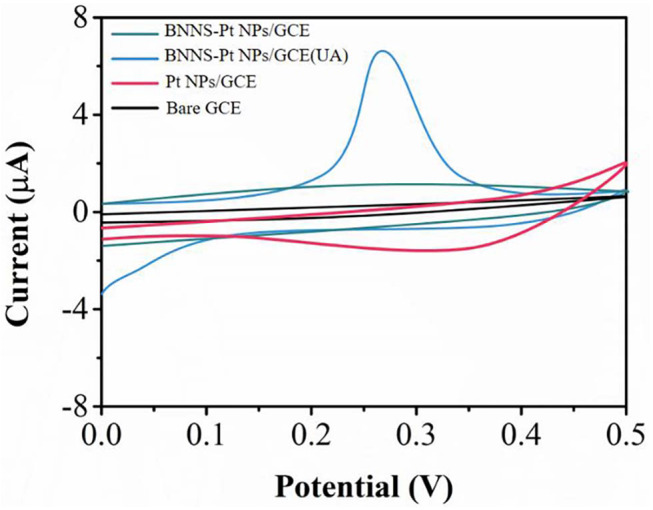
CV profiles of bare GCE, Pt NPs/GCE and BNNS-Pt NPs/GCE in 0.1 M PBS (pH 7.0) in the presence and the absence of 0.2 μM UA.

We tested the sensing performance of the proposed electrochemical sensor toward UA using ABS (pH4.5), PBS (pH 7.0), and Tris (pH9.5). The PBS showed the best performance. Therefore, we selected the PBS in this work. Figure 3 shows CV curves of 0.1 μM UA recorded at bare GCE and modified GCE in 0.1 M PBS. The UA shows no redox peaks at either GCE or BNNS/GCE. In contrast, BNNS-Pt NPs/GCE shows an oxidation peak due to the electrocatalytic activity of the Pt NPs. The Pt NPs may be applied as an excellent electrocatalyst for lowering the overpotential to overcome the interference compounds (Morsbach et al., 2014). In addition, the UA oxidation current at the BNNS-Pt NPs/GCE is larger than that of the Pt NPs/GCE, indicating the BNNS is an excellent substrate for Pt NP deposition. It can exhibit advanced electrocatalytic performance than chemically synthesized Pt NPs. The CV curve of BNNS-Pt NPs/GCE without UA was recorded as well. No obvious peak was noticed during the scan, suggesting the oxidation peak observed previously was caused by UA oxidation.

**Figure 4:**
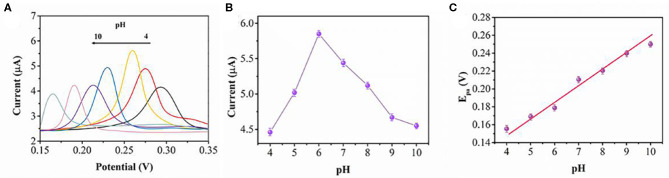
**(A)** SWV scans of BNNS-Pt NPs/GCE toward 0.2 μM UA at different pH values from 4.0 to 10.0. **(B)** The relationship of pH value and peak current. **(C)** Plot of pH vs. peak potential.

The effect of pH on the oxidation of 0.2 μM UA is shown in Figure 4A. The pH value of the PBS was changed by adding 0.1 M HCl or 0.1 M NaOH. We investigated UA oxidation in the pH range between 4.0 and 10.0 in PBS using SWV. Figure 4B shows the relationship between the oxidation current and pH conditions. The peak current increases when the pH increases. After pH 6, the peak current decreases with a further increase of pH. Therefore, pH 6 has been chosen for the optimum pH condition for UA detection. As shown in Figure 4C, the oxidation peak potential shifts along with the pH value. A linear expression can be deduced as: E_pa_(V)=(−0.0211) pH+0.0802 (*R* = 0.997). The number of electrons (n) in the overall reaction can be calculated as 2 for a totally irreversible diffusion-controlled process.

**Figure 5:**
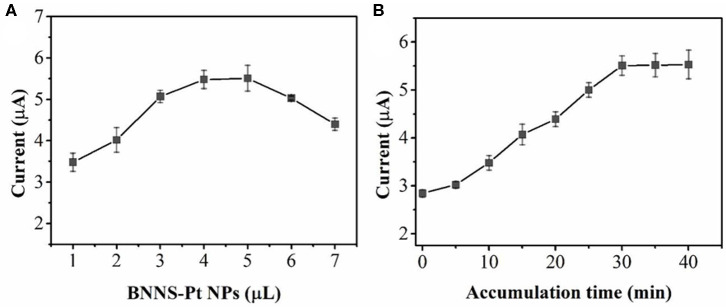
Effect of **(A)** the BNNS-Pt NPs content and **(B)** accumulation time toward 0.2 μM UA detection.

The effect of the amount of BNNS-Pt NPs on the UA oxidation has been optimized. As shown in Figure 5A, the oxidation peak current increases along with the BNNS-Pt NP loading increase from 1 to 5 μL. Further increases in the BNNS-Pt NPs will result in a lower current response due to the thicker modification layer and will consequently lower the electron transfer efficiency. Therefore, 5 μL of the BNNS-Pt NP dispersion was applied as electrode modifier. The accumulation process was further optimized for UA oxidation. As seen from Figure 5B, the peak response increases significantly from 0 to 30 min. Then, the peak currents remain steady with the further increase of accumulation time. Therefore, 30 min was chosen before sensing.

**Figure 6:**
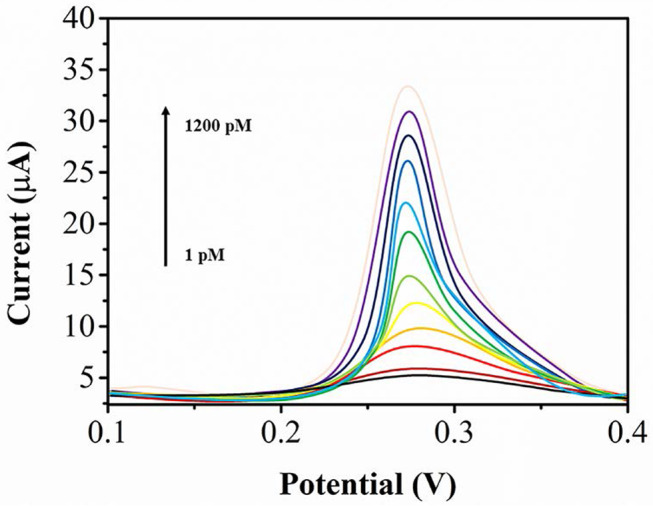
SWV profiles of UA at BNNS-Pt NPs/GCE from a to k: 1, 5, 20, 50, 100, 200, 300, 400, 500, 700, 900, 1,200 pM.

Under the optimum conditions, SWV was used for UA analysis. Figure 6 shows the SWV profiles of UA at BNNS-Pt NPs/GCE. The UA oxidation currents is linearly related its concentration from 1 to 1200 pM. The equations can be expressed as I_pa_ (μA) = 0.24221 c (nM) + 2.4454. The LOD can be calculated to be 0.5 pM (S/N=3).

The reproducibility of the proposed electrochemical sensor was investigated by the detection of 0.2 μM UA with eight individual detections. A RSD of 4.1% was recorded, indicating that the BNNS-Pt NP dispersion exhibits good reproducibility compared with previous BNNS based works (Fu et al., 2016, 2018a). The stability of the sensor was investigated by ten successive detections of 0.2 μM UA. The current remained at more than 85% after ten scans. The proposed electrochemical sensor could maintain more than 95% performance after 1 month storage.

**Figure 7:**
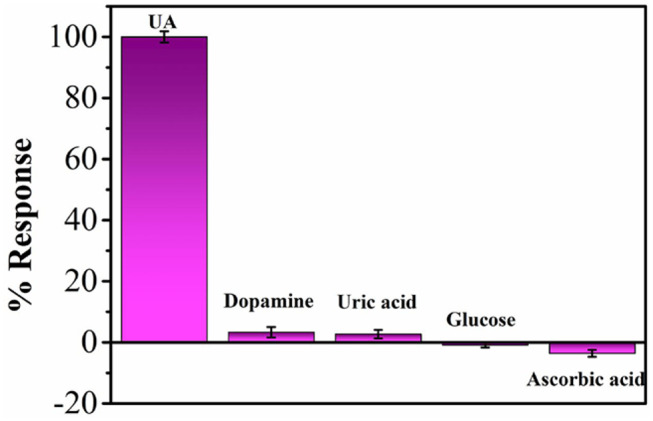
Percentage response of BNNS-Pt NPs/GCE for various interferences with respect to UA.

The anti-interference performance was investigated as well. The results show that 50-fold higher concentrations of common cations (Ca^2+^, Na^+^, K^+^, Zn^2+^) and anions (${\text{NO}}_{3}^{-}$, Cl^−^, ${\text{SO}}_{4}^{2-}$) show no changes of sensing. Ten-fold higher concentrations common species of dopamine, uric acid, glucose and ascorbic acid also show no changes of sensing (Figure 7). Therefore, the proposed BNNS-Pt NPs/GCE shows excellent selectivity for UA oxidation.

In this work, *Ligustri lucidum* fruit samples were used. The standard addition process was used and the results are showed in Table 1. The average UA concentration is found to be 0.397 μM in *Ligustri lucidum* fruit. An HPLC (Agilent 1100) method was also conducted for comparison.

**Table 1:** Detection of UA in *Ligustri lucidum* fruit and the recovery results.

**Sample**	**Found (μM)**	**HPLC (μMs)**	**Added (μM)**	**Found (μM)**	**Recovery (%)**	**RSD (%)**
1	0.399	0.387	0.5	0.885	98.44	3.12
2	0.413	0.405	0.5	0.901	98.69	2.65
3	0.427	0.425	0.5	0.922	99.46	3.22
4	0.350	0.352	0.5	0.841	99.94	4.05

## Conclusions

Deposition of Pt NPs on a BNNS surface can be used for UA electro-oxidation. The BNNS-Pt NPs nanocomposite exhibited electrocatalytic activity toward UA oxidation. Loading of Pt NPs on BNNS surface can provide more electrocatalytic sites by exposing individual Pt NPs to the analyte without agglomeration. The proposed UA sensing platform showed a linear detected from 1 to 1,200 pM, with a low LOD of 0.5 pM. In addition, the sensing platform has been successfully used for determining UA content in *Ligustri lucidum* fruit.

## Data Availability Statement

The raw data supporting the conclusions of this article will be made available by the authors, without undue reservation.

## Author Contributions

YF and JZ conducted the main experiments and wrote the paper. ZW did the material characterization and proofreading.

## Conflict of Interest

The authors declare that the research was conducted in the absence of any commercial or financial relationships that could be construed as a potential conflict of interest.

## References

[B1] Alam P. Al-Yousef H. M. Siddiqui N. A. Alhowiriny T. A. Alqasoumi S. I. Amina M.. (2018). Anticancer activity and concurrent analysis of ursolic acid, β-sitosterol and lupeol in three different Hibiscus species (aerial parts) by validated HPTLC method. Saudi Pharm. J. 26, 1060–1067. 10.1016/j.jsps.2018.05.015 30416363 PMC6218379

[B2] Azamat J. Sattary B. S. Khataee A. Joo S. W. (2015). Removal of a hazardous heavy metal from aqueous solution using functionalized graphene and boron nitride nanosheets: insights from simulations. J. Mol. Graph. Model. 61, 13–20. 10.1016/j.jmgm.2015.06.012 26186492

[B3] Baghayeri M. Amiri A. Alizadeh Z. Veisi H. Hasheminejad E. (2018a). Non-enzymatic voltammetric glucose sensor made of ternary NiO/Fe3O4-SH/para-amino hippuric acid nanocomposite. J. Electroanal. Chem. 810, 69–77. 10.1016/j.jelechem.2018.01.007

[B4] Baghayeri M. Amiri A. Maleki B. Alizadeh Z. Reiser O. (2018b). A simple approach for simultaneous detection of cadmium(II) and lead(II) based on glutathione coated magnetic nanoparticles as a highly selective electrochemical probe. Sens. Actuators B Chem. 273, 1442–1450. 10.1016/j.snb.2018.07.063

[B5] Baghayeri M. Maleki B. Zarghani R. (2014). Voltammetric behavior of tiopronin on carbon paste electrode modified with nanocrystalline Fe50Ni50 alloys. Mater. Sci. Eng. C 44, 175–182. 10.1016/j.msec.2014.08.023 25280694

[B6] Burhan H. Ay H. Kuyuldar E. Sen F. (2020). Monodisperse Pt-Co/GO anodes with varying Pt: Co ratios as highly active and stable electrocatalysts for methanol electrooxidation reaction. Sci. Rep. 10:6114. 10.1038/s41598-020-63247-6 32273553 PMC7145861

[B7] Chai J. Du X. Chen S. Feng X. Cheng Y. Zhang L.. (2015). Oral administration of oleanolic acid, isolated from *Swertia mussotii* Franch, attenuates liver injury, inflammation, and cholestasis in bile duct-ligated rats. Int. J. Clin. Exp. Med. 8, 1691–1702. 10.1155/2015/948376 25932098 PMC4402745

[B8] Demirkan B. Bozkurt S. Cellat K. Arikan K. Yilmaz M. Savk A.. (2020). Palladium supported on polypyrrole/reduced graphene oxide nanoparticles for simultaneous biosensing application of ascorbic acid, dopamine, and uric acid. Sci. Rep. 10:2946. 10.1038/s41598-020-59935-y 32076064 PMC7031288

[B9] Fu L. Chen G. Jiang N. Yu J. Lin C.-T. Yu A. (2016). *In situ* growth of metal nanoparticles on boron nitride nanosheets as highly efficient catalysts. J. Mater. Chem. A 4, 19107–19115. 10.1039/C6TA06409D

[B10] Fu L. Liu Z. Huang Y. Lai G. Zhang H. Su W. (2018a). Square wave voltammetric quantitative determination of flavonoid luteolin in peanut hulls and Perilla based on Au NPs loaded boron nitride nanosheets. J. Electroanal. Chem. 817, 128–133. 10.1016/j.jelechem.2018.04.009

[B11] Fu L. Wang A. Lai G. Su W. Malherbe F. Yu J.. (2018b). Defects regulating of graphene ink for electrochemical determination of ascorbic acid, dopamine and uric acid. Talanta 180, 248–253. 10.1016/j.talanta.2017.12.058 29332806

[B12] Fu L. Wang T. Yu J. Dai W. Sun H. Liu Z. (2017). An ultrathin high-performance heat spreader fabricated with hydroxylated boron nitride nanosheets. 2D Mater. 4:025047 10.1088/2053-1583/aa636e

[B13] Gao D. Li Q. Li Y. Liu Z. Liu Z. Fan Y.. (2007). Antidiabetic potential of oleanolic acid from *Ligustrum lucidum* Ait. Can. J. Physiol. Pharmacol. 85, 1076–1083. 10.1139/Y07-098 18066109

[B14] Gao L. Li C. Wang Z. Liu X. You Y. Wei H.. (2015a). Ligustri lucidi fructus as a traditional Chinese medicine: a review of its phytochemistry and pharmacology. Nat. Prod. Res. 29, 493–510. 10.1080/14786419.2014.954114 25244978

[B15] Gao R. Wang L. Yang Y. Ni J. Zhao L. Dong S.. (2015b). Simultaneous determination of oleanolic acid, ursolic acid, quercetin and apigenin in *Swertia mussotii* Franch by capillary zone electrophoresis with running buffer modifier. Biomed. Chromatogr. 29, 402–409. 10.1002/bmc.3290 25042239

[B16] Gharibi S. Bakhtiari N. Elham-Moslemee-Jalalvand Bakhtiari F. (2018). Ursolic acid mediates hepatic protection through enhancing of anti-aging biomarkers. Curr. Aging Sci. 11, 16–23. 10.2174/1874609810666170531103140 28558631

[B17] Hu B. Du Q. Deng S. An H.-M. Pan C.-F. Shen K.. (2014). *Ligustrum lucidum* Ait. fruit extract induces apoptosis and cell senescence in human hepatocellular carcinoma cells through upregulation of p21. Oncol. Rep. 32, 1037–1042. 10.3892/or.2014.3312 25017491

[B18] Hussain H. Green I. R. Ali I. Khan I. A. Ali Z. Al-Sadi A. M.. (2017). Ursolic acid derivatives for pharmaceutical use: a patent review (2012-2016). Expert Opin. Ther. Pat. 27, 1061–1072. 10.1080/13543776.2017.1344219 28637397

[B19] Karimi-Maleh H. Arotiba O. A. (2020). Simultaneous determination of cholesterol, ascorbic acid and uric acid as three essential biological compounds at a carbon paste electrode modified with copper oxide decorated reduced graphene oxide nanocomposite and ionic liquid. J. Colloid Interface Sci. 560, 208–212. 10.1016/j.jcis.2019.10.007 31670018

[B20] Karimi-Maleh H. Fakude C. T. Mabuba N. Peleyeju G. M. Arotiba O. A. (2019a). The determination of 2-phenylphenol in the presence of 4-chlorophenol using nano-Fe_3_O_4_/ionic liquid paste electrode as an electrochemical sensor. J. Colloid Interface Sci. 554, 603–610. 10.1016/j.jcis.2019.07.047 31330427

[B21] Karimi-Maleh H. Karimi F. Alizadeh M. Sanati A. L. (2019b). Electrochemical sensors, a bright future in the fabrication of portable kits in analytical systems. Chem. Rec. 10.1002/tcr.201900092. [Epub ahead of print]. 31845511

[B22] Li P. Liu A. Li Y. Yuan B. Xiao W. Liu Z.. (2019). Development and validation of an analytical method based on HPLC-ELSD for the simultaneous determination of rosmarinic acid, carnosol, carnosic acid, oleanolic acid and ursolic acid in rosemary. Molecules 24:323. 10.3390/molecules24020323 30658397 PMC6358743

[B23] Lim H. S. Oh J. W. Kim S. Y. Yoo M.-J. Park S.-D. Lee W. S. (2013). Anisotropically alignable magnetic boron nitride platelets decorated with iron oxide nanoparticles. Chem. Mater. 25, 3315–3319. 10.1021/cm401488a

[B24] Liu J. (1995). Pharmacology of oleanolic acid and ursolic acid. J. Ethnopharmacol. 49, 57–68. 10.1016/0378-8741(95)90032-2 8847885

[B25] Machado D. G. Neis V. B. Balen G. O. Colla A. Cunha M. P. Dalmarco J. B.. (2012). Antidepressant-like effect of ursolic acid isolated from *Rosmarinus officinalis* L. in mice: evidence for the involvement of the dopaminergic system. Pharmacol. Biochem. Behav. 103, 204–211. 10.1016/j.pbb.2012.08.016 22940588

[B26] Morsbach E. Brauns E. Kowalik T. Lang W. Kunz S. Bäumer M. (2014). Ligand-stabilized Pt nanoparticles (NPs) as novel materials for catalytic gas sensing: influence of the ligand on important catalytic properties. Phys. Chem. Chem. Phys. 16, 21243–21251. 10.1039/C4CP02993C 25188310

[B27] Nam S. Chang K. Lee W. Kim M. J. Hwang J. Y. Choi H. (2018). Structural effect of two-dimensional BNNS on grain growth suppressing behaviors in Al-matrix nanocomposites. Sci. Rep. 8, 1–12. 10.1038/s41598-018-20150-5 29371625 PMC5785491

[B28] Novotny L. Abdel-Hamid M. E. Hamza H. Masterova I. Grancai D. (2003). Development of LC-MS method for determination of ursolic acid: application to the analysis of ursolic acid in staphylea holocarpa hemsl. J. Pharm. Biomed. Anal. 31, 961–968. 10.1016/S0731-7085(02)00706-9 12684108

[B29] Orooji Y. Alizadeh A. ad Ghasali E. Derakhshandeh M. R. Alizadeh M. Asl M. S. (2019). Co-reinforcing of mullite-TiN-CNT composites with ZrB2 and TiB2 compounds. Ceram. Int. 45, 20844–20854. 10.1016/j.ceramint.2019.07.072

[B30] Pang Z. Zhi-yan Z. Wang W. Ma Y. Feng-ju N. Zhang X.. (2015). The advances in research on the pharmacological effects of fructus ligustri lucidi. BioMed Res. Int. 2015:281873. 10.1155/2015/281873 25874204 PMC4385624

[B31] Pironi A. M. de Araújo P. R. Fernandes M. A. Salgado H. R. N. Chorilli M. (2018). Characteristics, biological properties and analytical methods of ursolic acid: a review. Crit. Rev. Anal. Chem. 48, 86–93. 10.1080/10408347.2017.1390425 29039968

[B32] Rajski Ł. Beraza I. Gómez Ramos M. J. Ferrer C. Fernández-Alba A. R. (2020). Evaluation of segmented non-target data acquisition (SWATH/vDIA) in a QToF and QOrbitrap for pesticide residue analysis. Anal. Methods 12, 2027–2038. 10.1039/D0AY00290A

[B33] Ramos-Hryb A. B. Cunha M. P. Pazini F. L. Lieberknecht V. Prediger R. D. Kaster M. P.. (2017). Ursolic acid affords antidepressant-like effects in mice through the activation of PKA, PKC, CAMK-II and MEK1/2. Pharmacol. Rep. 69, 1240–1246. 10.1016/j.pharep.2017.05.009 29128805

[B34] Saraswat B. Visen P. K. Agarwal D. (2000). Ursolic acid isolated from Eucalyptus tereticornis protects against ethanol toxicity in isolated rat hepatocytes. Phytother. Res. 14, 163–166. 10.1002/(SICI)1099-1573(200005)14:3<163::AID-PTR588>3.0.CO 10815008

[B35] Saraswati S. Agrawal S. S. Alhaider A. A. (2013). Ursolic acid inhibits tumor angiogenesis and induces apoptosis through mitochondrial-dependent pathway in *Ehrlich ascites* carcinoma tumor. Chem. Biol. Interact. 206, 153–165. 10.1016/j.cbi.2013.09.004 24051192

[B36] Seo D. Y. Lee S. R. Heo J.-W. No M.-H. Rhee B. D. Ko K. S.. (2018). Ursolic acid in health and disease. Korean J. Physiol. Pharmacol. 22, 235–248. 10.4196/kjpp.2018.22.3.235 29719446 PMC5928337

[B37] Shamsadin-Azad Z. Taher M. A. Cheraghi S. Karimi-Maleh H. (2019). A nanostructure voltammetric platform amplified with ionic liquid for determination of tert-butylhydroxyanisole in the presence kojic acid. J. Food Meas. Charact. 13, 1781–1787. 10.1007/s11694-019-00096-6

[B38] Tahernejad-Javazmi F. Shabani-Nooshabadi M. Karimi-Maleh H. (2019). 3D reduced graphene oxide/FeNi3-ionic liquid nanocomposite modified sensor; an electrical synergic effect for development of tert-butylhydroquinone and folic acid sensor. Compos. Part B Eng. 172, 666–670. 10.1016/j.compositesb.2019.05.065

[B39] Wang T. Jin B. Jiao Z. Lu G. Ye J. Bi Y. (2014). Photo-directed growth of Au nanowires on ZnO arrays for enhancing photoelectrochemical performances. J. Mater. Chem. A 2, 15553–15559. 10.1039/C4TA02960G

[B40] Yan S. Huang C. Wu S. Yin M. (2010). Oleanolic acid and ursolic acid induce apoptosis in four human liver cancer cell lines. Toxicol. In Vitro 24, 842–848. 10.1016/j.tiv.2009.12.008 20005942

[B41] Yuliang W. Zejian W. Hanlin S. Ming Y. Kexuan T. (2015). The hypolipidemic effect of artesunate and ursolic acid in rats. Pak. J. Pharm. Sci. 28:77. 26004719

[B42] Zhang G. Qi Y. Lou Z. Liu C. Wu X. Chai Y. (2005). Determination of oleanolic acid and ursolic acid in cornel by cyclodextrin-modified micellar electrokinetic chromatography. Biomed. Chromatogr. 19, 529–532. 10.1002/bmc.475 15654724

